# Evaluation of radiographic and metabolic changes in bone metastases in response to systemic therapy with ^18^FDG-PET/CT

**DOI:** 10.1515/raon-2015-0012

**Published:** 2015-03-25

**Authors:** Bengul Gunalp, Ali Ozan Oner, Semra Ince, Engin Alagoz, Aslı Ayan, Nuri Arslan

**Affiliations:** 1 Gulhane Military Medical Academy and Faculty, Department of Nuclear Medicine, Ankara, Turkey; 2 Kocatepe University Medical Faculty, Department of Nuclear Medicine, Afyon, Turkey

**Keywords:** bone metastases, therapy response, ^18^FDG-PET/CT

## Abstract

**Background.:**

The aim of the study was to retrospectively evaluate radiographic and metabolic changes in bone metastases in response to systemic therapy with ^18^FDG-PET/CT and determine their roles on the evaluation of therapy response.

**Patients and methods.:**

We retrospectively evaluated radiographic and metabolic characteristics of bone metastases in 30 patients who were referred for the evaluation of response to systemic therapy with ^18^FDG-PET/CT. All patients underwent integrated ^18^FDG-PET/CT before and after treatment.

**Results.:**

The baseline radiographic patterns of the target lesions in responders group were lytic, sclerotic, mixed and CT negative; after treatment the radiographic patterns of all target lesions changed to a sclerotic pattern and attenuation increased (p = 0.012) and metabolic activity decreased (p = 0.012). A correlation was found between decreasing metabolic activity and increasing attenuation of the target lesions (r = −0.55) (p = 0.026). However, in nonresponders group, the baseline radiologic patterns of the target lesions were lytic, blastic, mixed and CT negative; after treatment all lytic target lesions remained the same and one CT negative lesion turned to lytic pattern and the attenuation of the target lesions decreased (p ± 0.12) and metabolic activity increased (p = 0.012). A correlation was found between increasing metabolic activity and decreasing attenuation (r = −0.65) (p = 0.032). An exception of this rule was seen in baseline blastic metastases which progressed with increasing in size, metabolic activity and attenuation.

**Conclusions.:**

This study shows that the metabolic activity of lesions is a more reliable parameter than the radiographic patterns for the evaluation of therapy response.

## Introduction

A significant fraction of patients with known malignancy develop osseous metastases and this usually mandates a radical change to the therapeutic approach. Early detection and evaluation of the treatment response is essential for making correct treatment decisions. Therefore, techniques are required to identify patients with active bone metastases and to monitor the treatment response in a timely manner.

Bone scintigraphy using technetium-99m-labeled diphosphonates has been the method of choice for the detection of bone metastases for many years but it is considered to be unsuitable for evaluating treatment response, because it has a poor specificity for detecting metastases, and positive lesions on a bone scan tend to remain positive even after effective treatment.^1^,^2^

On the basis of radiographic appearance, bone metastases are classified as osteolytic, osteoblastic (sclerotic), or mixed pattern. The sclerosis of a lytic component on computed tomography (CT) or plain radiographs is generally considered to suggest a response to treatment^1^–^4^, but if there are no baseline studies it is not possible to differentiate osteoblastic active metastases from sclerotic unviable metastases.

^18^F-fluorodeoxyglucose positron emission tomography/CT (^18^FDG-PET/CT) is widely used for the diagnosis and staging of various malignant tumors and evaluating responses to therapy.^5^–^7^ Abnormal bone ^18^FDG uptake by tumor cells is in proportion to levels of glucose metabolism. ^18^FDG is transported into tumor cells by glucose transporter proteins and phosphorylated by hexokinase to ^18^FDG-6-phosphate, which is retained in the malignant cells. Because FDG uptake is related to the metabolic activity of the tumor itself, ^18^FDG may detect metastases before bone destruction on a CT or osteoblastic healing on a bone scan.^8^–^10^

In previous investigations, it has been reported that in osteolytic metastases, ^18^FDG is more likely to be positive, while sclerotic metastases are less likely to be positive^11^–^14^, but changes of the radiographic and functional characteristics of the metastatic lesions during the treatment has not been investigated adequately. The clinical significance of ^18^FDG-positive/CT-negative lesions and CT-positive/^18^FDG-negative lesions pre and post-therapy conditions were unclear. In this retrospective study we tried to elucidate this issue determining functional and anatomic characteristics of bone metastases on pre and post-therapy conditions and their role on the evaluation of response to therapy.

## Patients and methods

### Patients

We retrospectively evaluated the radiographic and metabolic characteristics of bone metastases in 30 patients (mean age 58 years; age range 27–82) who were referred for the evaluation of response to systemic therapy with ^18^FDG-PET/CT between January 2013 and April 2014. All patients underwent integrated ^18^FDG-PET/CT before and after treatment. Two reviewers analyzed the images in consensus. Bone metastases in 15 patients with breast carcinoma, 10 patients with lung cancer, 2 patients with neuroendocrine tumor, 2 patients with lymphoma and 1 patient with sarcoma were included in this study (Table 1).

All patients received systemic therapy after baseline ^18^FDG-PET/CT imaging. Systemic therapy for breast carcinoma patients included the use of endocrine therapy, chemotherapy and/or biologic agents. A choice between them had been made depending on the status of hormone receptors (estrogen, progesterone receptor positive patients received anti-estrogen treatment with tamoxifen or letrazole), status of human epidermal growth factor 2 (HER2) overexpression (HER2 positive patients received biological treatment with trastuzumab or lapatinib). Chemotherapy was combined if patient hadn’t responded to endocrine or biological treatment. For metastatic lung carcinoma patients systemic therapy generally consisted of cytotoxic chemotherapy using cisplatin or carboplatin based doublet.

The response assessment was made according to biochemical, radiologic and clinical follow-up and ^18^FDG-PET/CT findings. Sixteen patients were classified as responders and 14 patients classified as non-responders or progressive disease. The morphologic appearance of the most prominent (target) lesion in each patient was classified as lytic, blastic (sclerotic), mixed or hypermetabolic bone lesion without CT findings (bone marrow) and lesion attenuation was measured as in Hounsfield units (HU) in axial CT images. The metabolic activity of the same lesion was calculated as the maximum standardized uptake value (SUV Max). The mean follow-up duration of the patients was 7 months, range 3 months to 18 months.

The institutional review board approved this study and for this type of study formal consent is not required (No. 50687469-1491-1311-13/1648.4-1472).

### Imaging

^18^FDG-PET/CT was performed prior to systemic therapy as a baseline study and after treatment (mean 28 days; range 24–32 days) in all patients. ^18^FDG-PET/CT images were acquired with an integrated PET/CT device (GE Discovery 690). Before ^18^FDG-PET/CT, patients fasted for at least 6 hours. All patients were tested to confirm that their glucose level was within the normal range [80–120 mg/dL (4.4–6.6 mmol/L)] before ^18^FDG administration. Before PET, unenhanced CT was performed from vertex to the mid-thigh according to a standardized protocol with the following settings: 120 kVp, 85 mA, Pitch 1.375 and slice thickness 3.75 mm.

Emission scans were obtained 60 minutes after intravenous administration of ^18^FDG (mean dose, 370 MBq; range 259–444 MBq). The acquisition time was 3 minutes per bed position in the two dimensional mode. Images were reconstructed with attenuation –weighted ordered –subset expectation maximization filter.

### Image interpretation and radiographic analysis

PET and CT images obtained in all standard planes were reviewed by two experienced nuclear medicine physicians. Images were analyzed visually and quantitatively. Only the lesion that exhibited the most prominent uptake was selected as the target lesion for response evaluation to the treatment. The exact anatomic location of the target lesion was identified on CT images and classified as lytic, blastic (sclerotic), mixed and no CT finding (bone marrow metastases without identifiable bone destruction on CT).

The change in CT attenuation (ΔAtt) (measured in Hounsfield units) in the region of interest (ROI) of the entire lesion before and after treatment was calculated with the following equation: ΔAtt = (Att_post_ - Att_pre_), where Att_pre_ and Att_post_ denote pre- and post-treatment attenuation, respectively.

The maximum standardized uptake value (SUV) was calculated with the following equation: SUV = *A*(ID/BW), where *A* is the decay-corrected mean activity in tissue (measured in millicuries per milliliter), ID is the injected dose of FDG (measured in millicuries), and BW is the patient’s body weight (measured in grams). Changes in SUV (ΔSUV) after treatment were calculated with the following equation: ΔSUV = (SUV_post_ - SUV_pre_), where SUV_pre_ and SUV_post_ denote pre and post-treatment SUV, respectively.

### Therapy response evaluation

Patients’ medical records and follow-up ^18^FDG-PET/CT findings were evaluated retrospectively. In patients who were designated as responders, the target lesion showed decreased uptake when compared with the same lesion depicted on baseline images and all biochemical, radiologic and clinical follow-up findings confirmed the response to therapy. In non-responders, a follow-up examination revealed substantially increased ^18^FDG uptake in the target lesion or additional new metastatic foci were identified on ^18^FDG-PET/CT images and all biochemical, radiologic and clinical findings confirmed a progression of the disease.

### Statistical analysis

Comparison of mean values between groups was performed with the Student t test. Spearman’s rho test was performed to investigate any correlation between attenuation (HU) and metabolic activity (SUV Max) of the lesions. P<0.05 was considered to indicate a significant difference. IBM SPSS statistics software (Version 21) was used for the statistical analysis.

## Results

The radiographic pattern of the target lesions on the baseline PET/CT images was classified as lytic in 13 (43%) patients, blastic (sclerotic) in 7 (23%) patients, mixed in 3 (10%) patients and no CT abnormality on target lesion (bone marrow metastases) in 7 (23%) patients.

### Responders group

**FIGURE 1.**
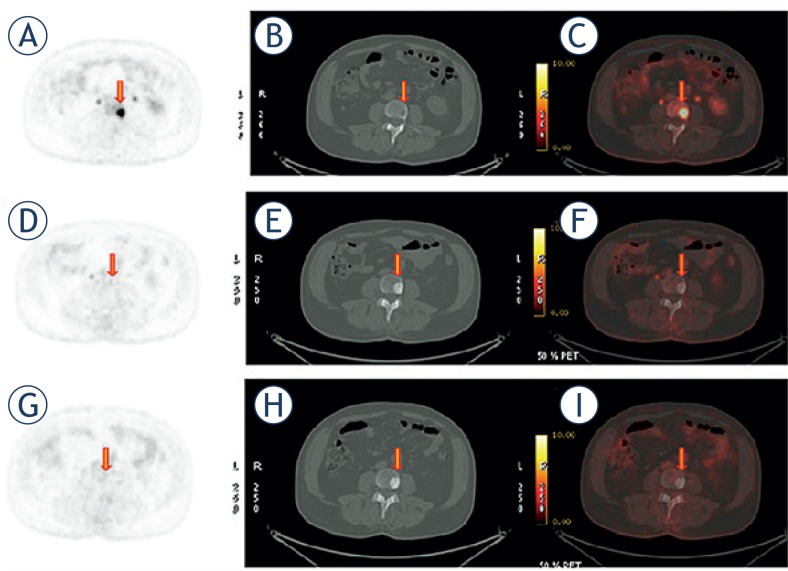
Baseline lytic lesion is healing with sclerosis. Baseline transaxial **(A)** ^18^FDG PET, **(B)** CT, and ^18^FDG-PET/CT images 65 years old man with lung carcinoma show lytic bone metastasis in vertebra (arrow). Maximum standardized uptake value (SUV) Max: 14.8, Hounsfield units (HU): 58; **(D)**, **(E)**, **(F)** 9 months after therapy lesion metabolic activity decreased SUV Max: 1.6, attenuation increased HU: 780; **(G)**, **(H)**, **(I)** 12 months after therapy. Inactive sclerotic metastasis with no metabolic activity and sclerotic appearance on CT with increased attenuation HU: 934.

**FIGURE 2.**
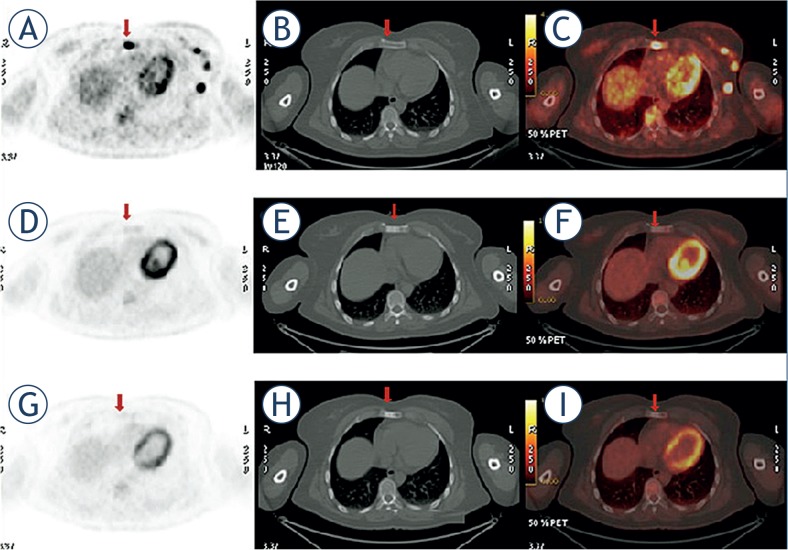
Baseline CT-negative ^18^FDG-positive bone marrow metastasis is becoming sclerotic (CT-positive) while decreasing metabolic activity (^18^FDG-negative) as a response to therapy. Baseline transaxial **(A)** ^18^FDG- PET, **(B)** CT, and ^18^FDG-PET/CT images in 45 years old woman with breast carcinoma show bone marrow metastasis in sternum (arrow) without corresponding CT abnormalities. Maximum standardized uptake value (SUV Max): 11, Hounsfield units (HU): 73; **(D)**, **(E)**, **(F)** 6 months after therapy lesion metabolic activity decreased SUV Max: 2.7, attenuation increased HU: 551; **(G)**, **(H)**, **(I)** 9 months after therapy. Inactive sclerotic metastasis with no increase metabolic activity and sclerotic appearance on CT with increased attenuation HU: 693.

There were 16 (53%) patients whose metabolic activity of the target lesion decreased after treatment and clinical follow-up confirmed the therapy response. The baseline radiographic patterns of the target lesions were lytic in 6 (37%) patients, blastic (sclerotic) in 5 (31%) patients, mixed in 2 (13%), bone marrow in 3(19%) and the mean attenuation was HU = 190 ± 137; the mean metabolic activity was SUV Max = 8.78 ± 3.09; after treatment the radiographic patterns of all target lesions turned to a sclerotic pattern, as shown in Figures 1, 2, attenuation increased (mean HU = 622 ± 273) (p = 0.012) and metabolic activity decreased (SUV Max: 2.92 ± 1.07) (p = 0.012). A negative correlation was found between decreasing metabolic activity (SUV Max) and increasing attenuation (HU) of the target lesions (r = −0.55) (p = 0.026). Three patients with increased metabolic activity on PET and any corresponding radiographic pathologic finding on CT turn to sclerotic lesion after treatment. Bone metastases of all tumor types with different radiological patterns on baseline CT scan showed sclerotic pattern on post-therapy scan if therapy response was achieved.

### Non-responders group

**FIGURE 3.**
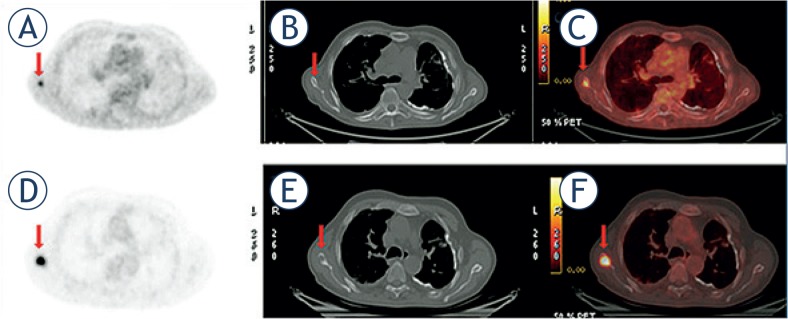
Progression of CT-negative ^18^FDG-positive bone marrow metastasis with becoming lytic lesion on CT. Pre-treatment transaxial **(A)** ^18^FDG- PET, **(B)** CT, and **(C)** ^18^FDG-PET/CT images in 67 years old man with lung carcinoma show hypermetabolic bone metastasis in scapula (arrow) without any evidence on CT. Maximum standardized uptake value (SUV Max): 2.6, Hounsfield units (HU): 165; **(D)**, **(E)**, **(F)** 6 months after therapy bone metastasis did not respond to the therapy and the disease progressed. The lesion became lytic, its attenuation decreased HU: 84 and metabolic activity increased SUV Max: 19.9.

There were 14 (47%) patients whose metabolic activity of the target lesion increased after therapy and progression of disease was confirmed by biochemical, radiologic and clinical findings. The baseline radiographic patterns of the target lesions were lytic (n = 7), blastic (n = 2), mixed (n = 1), CT negative (bone marrow) (n = 4), the mean attenuation was HU = 349 ± 290 and mean metabolic activity was SUV Max = 7.6 ± 2.95; after treatment radiographic patterns of all lytic target lesions remained the same and one bone marrow lesion turned to a lytic pattern and attenuation of the target lesions decreased (HU 168 ± 212) (p = 0.12) and metabolic activity increased (SUV Max:11.0 ± 5.3) (p = 0.012) as shown in Figure 3. A negative correlation was found between increasing metabolic activity and decreasing attenuation (r = −0.65) (p = 0.032).

**FIGURE 4.**
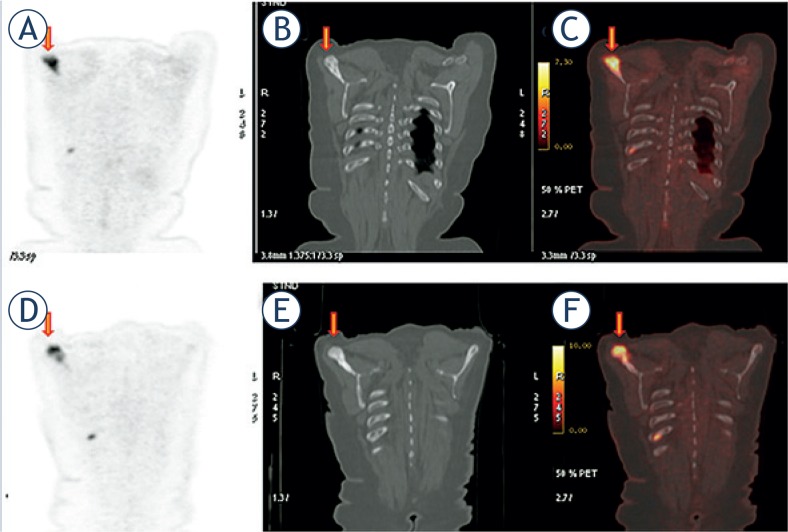
Progression of blastic metastasis with increasing metabolic activity and density. Pre-treatment coronal **(A)** ^18^FDG-PET, **(B)** CT, and **(C)** ^18^FDG-PET/CT images in 57 years old woman with breast carcinoma show blastic metastasis in scapula (arrow). Maximum standardized uptake value (SUV Max): 7.5, Hounsfield units (HU): 917; **(D)**, **(E)**, **(F)** 4 months after therapy bone metastasis did not respond to the therapy and the disease progressed. The lesion metabolic activity increased SUV Max: 11.1 and attenuation increased HU: 1056

There were 3 (21%) patients with blastic and mixed metastases on their baseline study that progressed with increasing in size, metabolic activity and attenuation as shown in Figure 4. A summary of the results is illustrated in Table 2.

## Discussion

Our results show that an increase in attenuation and a decrease in SUV of bone metastases after systemic treatment are associated with therapy response as reported in previous studies.^15^,^16^ All morphologic types of baseline metastatic lesions turned to sclerotic lesions if a therapy response was achieved. On the other hand, if a therapy response was not achieved different patterns were recognized which were not described in previous studies. If therapy response was not achieved while all baseline lytic lesions remained as lytic and CT-negative bone marrow lesions turned to lytic lesions but blastic lesions remained as blastic with increased metabolic activity, density and size. These findings were found to be highly correlated with the pathophysiology of bone metastases. In osteolytic bone metastases, tumor cells release humoral factors that stimulate osteoclastic activity and osteoclasts start to break down bone. Bone resorption results in the release of growth factors that stimulate tumor cell growth. In osteoblastic bone metastases, tumor cells secrete growth factors that stimulate the activity of osteoblasts. Excessive new bone formation occurs around tumor-cell deposits. Osteoblastic activation releases unidentified osteoblastic growth factors that also stimulate tumor cell growth.^17^ Although we had very a few numbers of patients in non-responders group with blastic metastases our findings also confirmed continuing excessive new bone formation if therapy response was not achieved.

Our findings suggest that ^18^FDG uptake reflects the tumor activity of bone metastases independent of the radiographic characteristics. Since both blastic metastases and sclerotic lesions show increased attenuation on CT, it is not possible to differentiate them based on their CT characteristics. ^18^FDG-PET/CT enables the clinician to differentiate metabolically active tumor tissue in “blastic metastases” from scar tissue in sclerotic lesions. The radiographic changes vary greatly among individual patients and do not seem to correlate with the presence of an active tumor. This study also shows that sequential ^18^FDG-PET/CT can provide vital information in monitoring the response of bone metastases to therapy.

In this study we observed “early marrow-based” metastases in 7 (23%) patients with increased metabolic activity confined to bone marrow without corresponding morphologic changes on CT. This finding is consistent with previous reports which documented that ^18^FDG-PET/CT detects bone metastases earlier than CT when there are a substantial number of metabolically active tumor cells present in bone marrow but still tumor invasion of the bone matrix doesn’t occur.^18^,^19^

In the previous studies ^18^FDG-PET has been found less sensitive than CT and bone scan in the detection of sclerotic metastases, however, most of the patients included in these studies had been treated with a systemic therapy^20^–^24^, and it has been suggested that osteolytic bone metastases may become sclerotic after effective treatment.^25^ ^18^FDG-PET/CT combines both metabolic and anatomic information on the same image and provides more accurate assessment of bone metastases than does either PET and CT alone.

Hybrid ^18^FDG-PET/CT provides us with a simultaneous comparison of functional and morphologic changes in bone metastases during the therapy and our study showed that if therapy response is achieved the most of the FDG positive bone metastases on pre-treatment study are becoming ^18^FDG negative and sclerotic on CT. Our study confirmed that pre-treatment ^18^FDG positive, post-treatment ^18^FDG negative sclerotic lesions belong to scar tissue which is developing during the healing process of bone metastases.

## Conclusions

This study shows radiographic patterns of bone metastases on CT changing during the therapy and the therapy response cannot be evaluated with the radiographic appearance of the lesion on CT. PET/CT has been found more sensitive and specific than CT both in detecting and evaluating the therapy response of bone metastases.

## Figures and Tables

**TABLE 1.** Baseline characteristics of patients

**Characteristic**	**Value**
Mean age (y)	58 ± 13 (27–82)^*^
Sex	
Female	21 (70)^**^
Male	9 (30)
Type of cancer	
Breast cancer	15 (50)
Lung cancer	10 (33)
Neuroendocrine tumor	2 (6)
Lymphoma	2 (6)
Sarcoma	1 (3)

* Data are mean ± standard deviation. Data in parentheses are range;

** Data are numbers of patients and data in parentheses are percentages

**TABLE 2.**
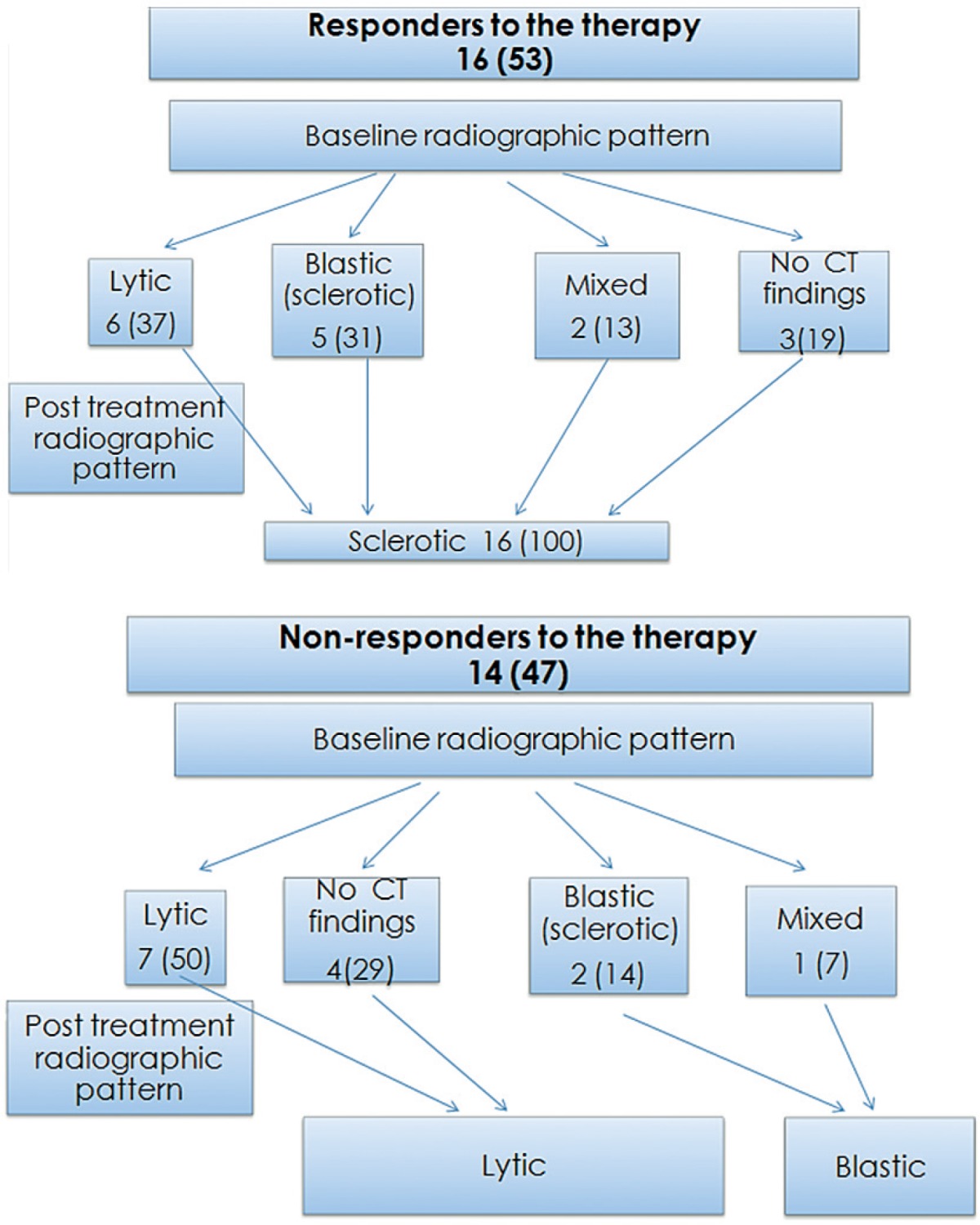
Summary of the results

Data are numbers of patients and data in parentheses are percentages
